# A Heuristic Approach Based on Clarke-Wright Algorithm for Open Vehicle Routing Problem

**DOI:** 10.1155/2013/874349

**Published:** 2013-12-05

**Authors:** Tantikorn Pichpibul, Ruengsak Kawtummachai

**Affiliations:** Faculty of Business Administration, Panyapiwat Institute of Management, 85/1 Moo 2, Chaengwattana Road, Bangtalad, Pakkred, Nonthaburi 11120, Thailand

## Abstract

We propose a heuristic approach based on the Clarke-Wright algorithm (CW) to solve the open version of the well-known capacitated vehicle routing problem in which vehicles are not required to return to the depot after completing service. The proposed CW has been presented in four procedures composed of Clarke-Wright formula modification, open-route construction, two-phase selection, and route postimprovement. Computational results show that the proposed CW is competitive and outperforms classical CW in all directions. Moreover, the best known solution is also obtained in 97% of tested instances (60 out of 62).

## 1. Introduction

The open vehicle routing problem (OVRP) was firstly solved by Sariklis and Powell [1] in their paper on distribution management problems. The characteristics of OVRP are similar to the capacitated vehicle routing problem (CVRP), which can be described as the problem of determining a set of vehicle routes to serve a set of customers with known geographical coordinates and known demands. A route represents a sequence of locations that a vehicle must visit. The distances between customer locations and between them and the depot are calculated or known in advance. For each route, the vehicle departs from the depot and returns to the depot after completing the service. The CVRP involves a single depot, a homogeneous fleet of vehicles, and a set of customers who require delivery of goods from the depot. The objective of the CVRP is to construct a feasible set of vehicle routes that minimizes the total traveling distance and/or the total number of vehicles used. Furthermore, the route must satisfy the constraints that each customer must be visited once, the demands of customers are totally satisfied, and the vehicle capacity is not exceeded for each route. In contrast, in the OVRP the companies either do not have their own vehicle available or the vehicles are inadequate to serve their customers. In this situation, the subcontracted vehicles will be hired from logistics outsourcing companies. Therefore, the transportation cost only depends on the traveling distance from depot to customers in which vehicles do not return to the depot and the maintenance cost does not occur. In another situation, the vehicles may return to the depot by following the same route in reverse order to collect items from the customers. The real-world case studies of OVRP are presented including a train plan model for British Rail freight services through the Channel Tunnel [2], the school bus routing problem in Hong Kong [3], the distribution of fresh meat in Greece [4], the distribution of a daily newspaper in the USA [5, 6], a lubricant distribution problem in Greece [7], and a mines material transport vehicle routing optimization in China [8].

**Figure 1:**
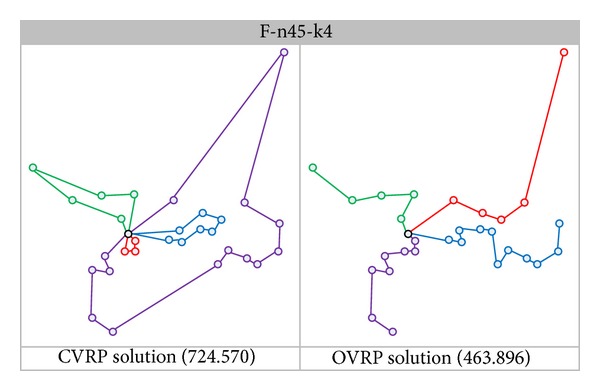
The difference of best known solutions between CVRP and OVRP.

The CVRP is one of the most important and widely studied problems in the area of combinatorial optimization. It comprises the traveling salesman problem (TSP) and the bin packing problem. The main distinction between OVRP and CVRP is that in CVRP each route is TSP which requires a Hamiltonian cycle [9], but in OVRP each route is a Hamiltonian path. Held and Karp [10] and Miller and Thatcher [11] have shown that the TSP is classified as NP-hard (Non-deterministic Polynomial-time hard) problem. In addition, the Hamiltonian path has also been shown to be NP-hard [12]. Besides, the CVRP and OVRP are NP-hard [13–15]. In Figure 1, the best known solution for the OVRP is very different from the CVRP, and we also refer to Syslo et al. [12] that deleting the largest edge from a minimum Hamiltonian cycle does not necessarily yield the minimum Hamiltonian path in the network. The CVRP has attracted many researchers since Dantzig and Ramser [16] proposed the problem in 1959. Many efficient heuristics were presented to solve the CVRP. Conversely, from the early 1980s to the late 1990s, OVRP received very little attention in the operations research literature compared to CVRP. However, since 2000, several researchers have used various heuristics to solve OVRP, such as a tabu search [14, 17, 18], an ant colony system [19], a variable neighborhood search [20], a particle swarm optimization [21], and a genetic algorithm [8].

The CW was proposed by Clarke and Wright [22] who introduced the savings concept which is based on the computation of savings for combining two customers into the same route. The CW is a widely known heuristic for solving the vehicle routing problem (VRP), and the applications of CW have continued since it was proposed in 1964. Improvements to the CW solution include proposed new parameters to the Clarke-Wright formulation composed of the nearest terminal *k* for solving multidepot VRP [23], deleting *c*_*j*,1_ for solving OVRP [24], an estimate of the maximum savings value *s*_max⁡_, and a penalty multiplier *α* for solving VRP with backhauls [25], route shape *λ* for solving CVRP [26, 27], weight *μ* for asymmetric solving CVRP [28], the customer demand *ν* for solving CVRP [29], and the cosine value of polar coordinate angles of customers with the depot cos⁡⁡*θ*_*i*,*j*_ for solving CVRP [30]. Second is improvements to the CW solution by proposed new probabilistic approaches to the CW procedure composed of the Monte Carlo simulation, cache, and splitting techniques for solving CVRP [31, 32], the two-phase selection and route postimprovement for solving CVRP [33, 34]. Based on our review, there are very few works available in the literatures to modify the CW for solving OVRP (only Bodin et al. [24]). Therefore, this is our major contribution to improve the CW solution by using our simple, efficient, and competitive approach. In the proposed CW, we have modified the parallel version of CW to deal with OVRP and have combined this with a route postimprovement procedure which refers several neighborhood structures from the works of Subramanian et al. [35] and Groër et al. [36]. Moreover, the numerical experiment of CW for solving OVRP benchmark instances is also presented.

## 2. The Proposed Clarke-Wright Algorithm

**Figure 2:**
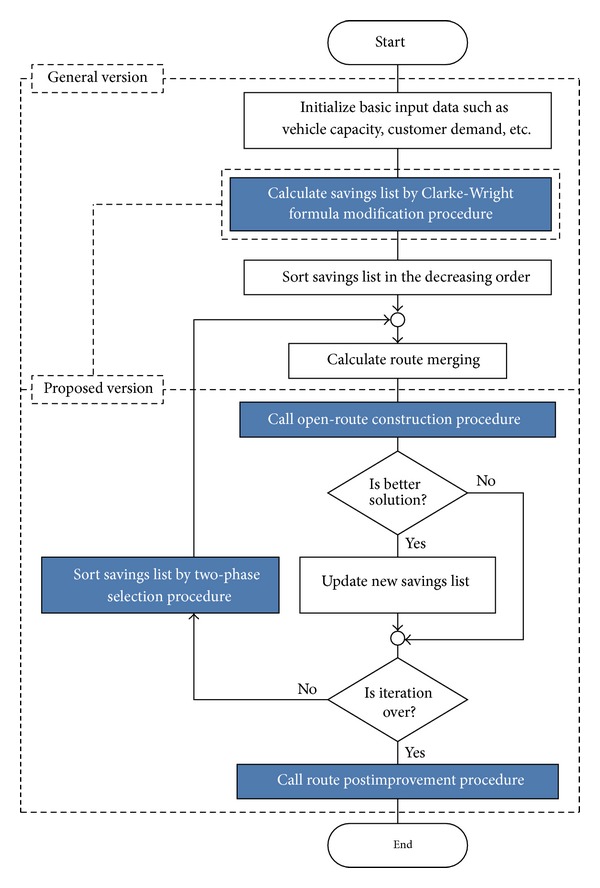
The flowchart of the proposed CW.

Because CW is a heuristic algorithm, it cannot guarantee the best solution. Therefore, we introduce the modified version of the Clarke-Wright algorithm in which the parallel version of CW is implemented since it usually generates better results than the corresponding sequential version [13, 37]. The flowchart of the proposed CW is given in Figure 2. First, the Euclidean distance matrix (*c*_*i*,*j*_) is calculated with the following equation:

(1) $$\begin{array}{c} c_{i,j}=\sqrt{{\left(x_{i}-x_{j}\right)}^{2}+{\left(y_{i}-y_{j}\right)}^{2}}, \end{array}$$

where *x*_*i*_, *y*_*i*_ and *x*_*j*_, *y*_*j*_ are the geographical locations of customer *i* and *j*. Second, the savings value between customer *i* and *j* is calculated as

(2) $$\begin{array}{c} s_{i,j}=c_{1,j}-c_{i,j}, \end{array}$$

where *c*_1,*j*_ is the traveling distance between depot and customer *j* and *c*_*i*,*j*_ is the traveling distance between customer *i* and *j*. Equation (2) is modified by Bodin et al. [24] from the Clarke-Wright formulation which is shown in (3). After calculation, all savings values are collected in the savings list as follows:

(3) $$\begin{array}{c} s_{i,j}=c_{1,i}+c_{j,1}-c_{i,j}. \end{array}$$

**Figure 3:**
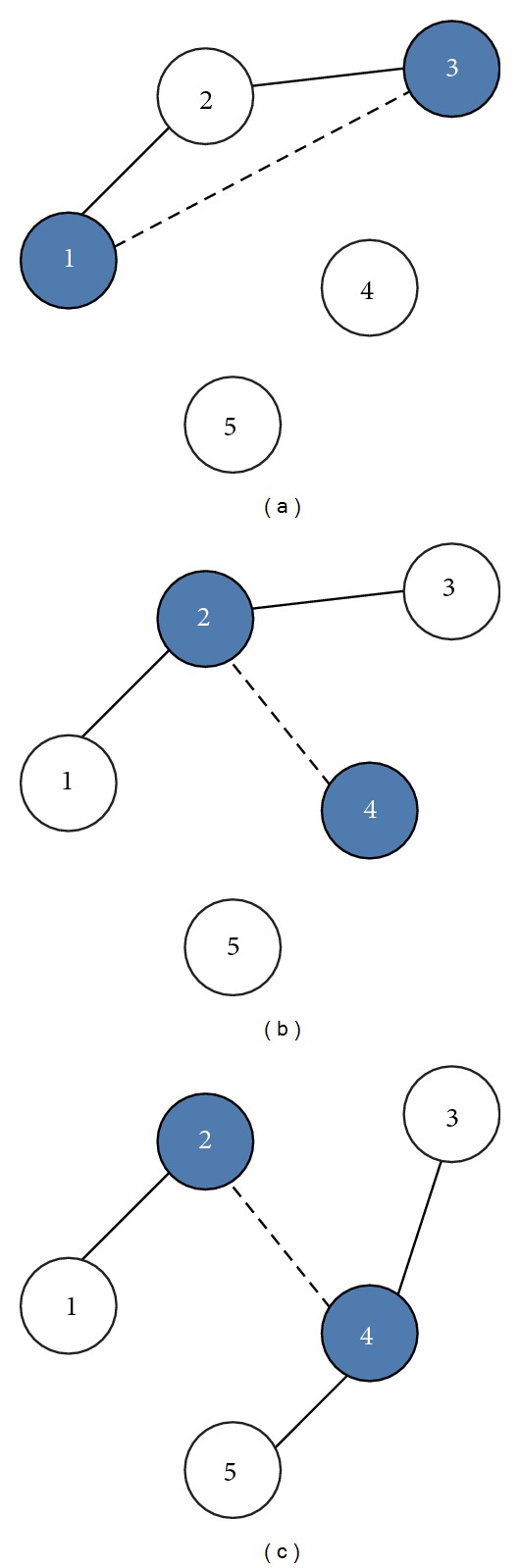
The route constraints of route merging procedure.

Third, the values in the savings list are sorted in decreasing order. Finally, the route merging procedure starts from the top of the savings list (the largest *s*_*i*,*j*_). Both customers *i* and *j* will be combined into the same route if the total demand does not exceed vehicle capacity and no route constraints exist. Each condition for route constraints is described by three cases of five customers as shown in Figure 3. In Figure 3(a), (1 nor 3) have already been assigned to a route (1-2-3). In Figure 3(b), exactly one of the two customers (2 or 4) has already been included in an existing route (1-2-3) and customer (2) is not interior to that route (a customer is interior to a route if it is not adjacent to the depot in the order of traversal of customers). In Figure 3(c), both customers (2 and 4) have already been included in two different existing routes (1-2 and 3-4-5), and customer (4) is also interior to its route (3-4-5). The route merging procedure is repeated until no feasible merging in the savings list is possible. Furthermore, in case of nonrouted customers, each is assigned by a route that starts at the depot, visits the unassigned customer, and returns to the depot.

The proposed CW is an iterative improvement approach designed to find the global optimum solutions. It has been presented in four procedures consisting of Clarke-Wright formula modification, open-route construction, two-phase selection, and route postimprovement. The details of these procedures are shown below.

### 2.1. The Clarke-Wright Formula Modification Procedure

Due to the latest improvement of CW for solving VRP that we mentioned in the literatures above, many authors proposed new parameters which we applied to this procedure. Gaskell [26] and Yellow [27] presented a route shape (*λ*) parameter which controls the relative significance of direct arc between two customers. Their proposed savings formula is as follows:

(4) $$\begin{array}{c} s_{i,j}=c_{1,i}+c_{j,1}-\lambda c_{i,j}. \end{array}$$

According to (2), we also modified (4) by deleting *c*_*j*,1_ for solving OVRP with the following equation:

(5) $$\begin{array}{c} s_{i,j}=c_{1,j}-\lambda c_{i,j}. \end{array}$$

The parameter could be varied as studied by Altinel and Öncan [29]. They used a simple enumerative approach to produce 8820 different solutions (*λ* ∈ [0.1,2], *μ* ∈ [0,2], *ν* ∈ [0,2]). After that the best solution will be chosen. In order to avoid time-consuming iterative solutions, we have therefore applied only single parameter (*λ*) to this procedure.

### 2.2. The Open-Route Construction Procedure

**Figure 4:**
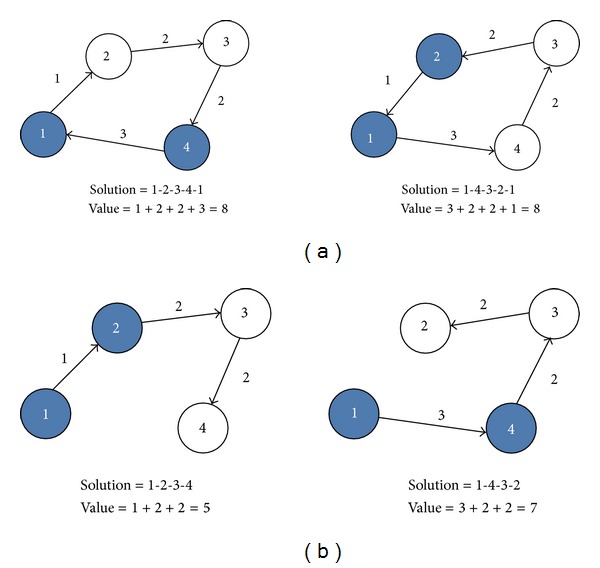
The two possible CVRP and OVRP solutions.

In order to solve CVRP by the CW after the route merging procedure, this procedure is needed to create the solution. Each route has to construct for the close route (Hamiltonian cycle) by assigning the first customer who starts at the depot, and the last customer who returns to the depot. As shown in Figure 4(a), the Euclidean distance represented in this paper is symmetric. Therefore, the two possible CVRP solutions (1-2-3-4-1 and 1-4-3-2-1) are similar. In contrast, in OVRP each route has to construct for the open route (Hamiltonian path) by only assigning the first customer who starts at the depot. As shown in Figure 4(b), the two possible OVRP solutions (1-2-3-4 and 1-4-3-2) are very different. Consequently, in this procedure, the two possible OVRP solutions are constructed, and then the best solution will be selected as the OVRP solution.

### 2.3. The Two-Phase Selection Procedure

**Figure 5:**
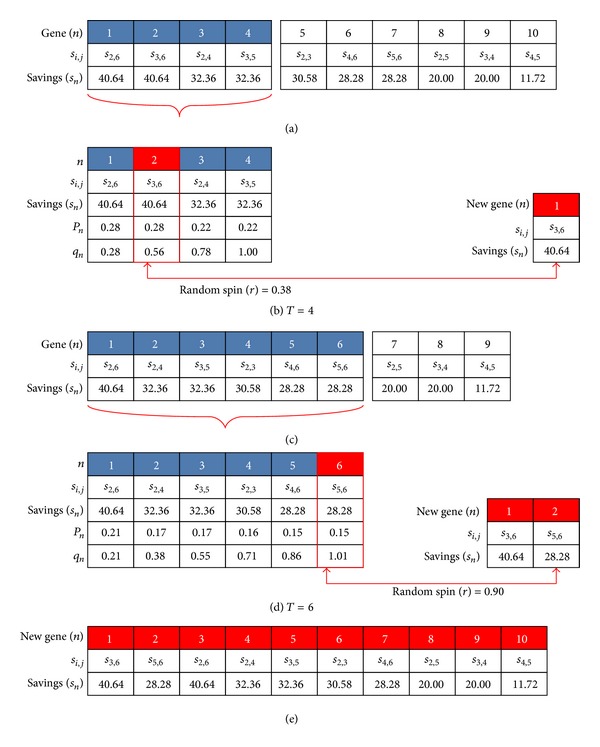
The example of the two-phase selection procedure.

After the CW solution is produced from a standard savings list generated by sorting the savings values in the decreasing order, this savings list will be regenerated as a new one by sorting the savings values randomly with probability. Pichpibul and Kawtummachai [34] introduced the two-phase selection procedure for CVRP. In this paper, we adjust this procedure to deal with an OVRP by based on an operation of the genetic algorithm [38].  Figure 5(a) shows a genetic representation of chromosomes for ten savings values. The savings list is represented by one chromosome, and each gene represents the savings value between customers *i* and *j*. In the first iteration, the chromosome is the savings list sorted by the decreasing order, but in next iteration the chromosome is the savings list derived from the best one. In Figure 5(b), we select one gene from the top four genes (*T* = 4) in the chromosome by fitness proportionate selection or roulette wheel selection. Here *T* is tournament size which is a random number between three and six. In order to create a roulette wheel, the selection probability (*p*_*n*_) and cumulative probability (*q*_*n*_) with savings value (*s*_*n*_) for each gene (*n*) are calculated using the following equations:

(6) $$\begin{array}{c} p_{n}=\frac{s_{n}}{\sum_{i\in T}s_{i}}\;\text{for}\;n\in T,\\ q_{n}=\sum_{i\in n}p_{i}\;\text{for}\;n\in T. \end{array}$$

After that, we spin the wheel with a random number (*r* = 0.38) from the range between 0 and 1. The one savings value (*s*_2_) will be selected to be a gene of a new chromosome by considering *r* and *q*_*n*_. If *r* ≤ *q*_1_, then select the first savings value *s*_1_; otherwise, select the *n*th savings value *s*_*n*_  (2 ≤ *n* ≤ *T*) such that *q*_*n*−1_ < *r* ≤ *q*_*n*_. The selected gene is removed from the chromosome which leaves nine savings values as shown in Figure 5(c).  Figure 5(d) shows the same selection process in the next iteration with parameters *T* = 6 and *r* = 0.90. Therefore, this procedure will be executed until the last gene of the chromosome is selected to be a gene of the new chromosome which is shown in Figure 5(e).

When the new chromosome which represents the new savings list is generated, it is calculated by the route merging procedure and the open-route construction procedure to produce a new OVRP solution. After we compare two solutions, the new chromosome will replace the previous chromosome only if the new solution is better than the previous one. This acceptance criterion is referred to a basic variable neighborhood search which is to accept only improvements [39]. Our approach is continued until the stopping criterion, which is the number of global iterations for two-phase selection, is satisfied.

### 2.4. The Route Postimprovement Procedure

In order to find any further improvements for the best solution found when the stopping criterion of the two-phase selection procedure is satisfied, we have developed the route postimprovement procedure to generate different routes in our best solution. In order to explore the whole neighborhood of our best solution, we focus on the order of customers in single route called intraroute and multiple routes called interroute. The neighborhood structures that we used are several well-known move operators found in the works of Subramanian et al. [35] and Groër et al. [36] including shift moves (1-0, 2-0, 3-0), swap moves (1-1, 2-1, 2-2), and *λ*-opt moves (*λ* ∈ {2,3}). The shift moves remove customers and insert them in another place. The swap moves select customers and exchange them. The *λ*-opt moves remove edges between customers and replace them with new edges. Our scheme is adapted from the local search strategy found in Çatay [40] by first applying a local neighborhood search to improve our best solution in each route. Then, a larger neighborhood search is applied across each pair between routes, respectively, by using all eight move operators with equal probability. This procedure is repeated until the stopping criterion, which is the number of consecutive iterations without any improvements in the best found solution, is satisfied.

## 3. Computational Results

The proposed CW was coded in Visual Basic 6.0 on an Intel Core i7 CPU 860 clocked at 2.80 GHz with 1.99 GB of RAM under Windows XP platform. The numerical experiment used five well-known data sets of Euclidean benchmarks (composed of 62 instances) of the OVRP consisting of Augerat et al. [41] in data sets A, B, and P, Christofides and Eilon [42] in data set E, and Fisher [43] in data set F. The input data is available online at http://www.branchandcut.org/ (last access 1/2010). The best known solutions which are available online at http://www.hha.dk/~lys/ (last access 7/2011) are obtained by a branch-and-cut algorithm from the work of Letchford et al. [44]. In our approach, some parameters have to be preset before the execution as shown in Table 1.  Table 2 describes the development of the proposed CW in detail.

The benchmark problem sizes that we highlighted in this paper are classified as small-scale (less than 50 customers) and medium-scale (between 51 to 100 customers) with different features, for example, uniformly and not uniformly dispersed customers, clustered and not clustered, with a centered or not centered depot. All problems also include capacity constraints and minimum number of vehicles used restrictions. The first benchmark in data sets A, B, and P was proposed by Augerat et al. [41]. For the instances in data set A, both customer locations and demands are randomly generated. The customer locations in data set B are clustered instances. The modified version of other instances is data set P. In these data sets, the problem ranges in size from 16 to 69 customers including the depot. The second benchmark in data set E was proposed by Christofides and Eilon [42]. In this data set, the customers are randomly distributed in the plane and the depot is either in the center or near to it. The problem ranges in size from 22 to 101 customers including the depot. The third benchmark in data set F is the real-life problem given by Fisher [43]. Instances F-n45-k4 and F-n135-k7 represent a day of grocery deliveries from the Peterboro and Bramalea, Ontario terminals, respectively, of National Grocers Limited. Instance F-n72-k4 represents the delivery of tires, batteries, and accessories to gasoline service stations. The depot is not centered in both instances. The problem ranges in size from 45 to 135 customers including the depot. We discuss each benchmark problem in which the percentage improvement between CW solution (cws) and obtained solution (obs) is calculated as follows:

(7) $$\begin{array}{c} \text{Percentage improvement}=\left(\frac{\text{cws}-\text{obs}}{\text{cws}}\right)\times 100. \end{array}$$

Moreover, the percentage deviation between obtained solution (obs) and the best known solution (bks) is also calculated as follows:

(8) $$\begin{array}{c} \text{Percentage deviation}=\left(\frac{\text{obs}-\text{bks}}{\text{bks}}\right)\times 100. \end{array}$$

The computational results for OVRP benchmark instances of Augerat et al. [41], Christofides and Eilon [42], and Fisher [43] are reported in Tables 3–5. We do not only consider the improvements of our solutions over CW solutions as shown in Table 3, but also show the performance of our solutions by comparing the proposed CW with the algorithms for OVRP as shown in Tables 4 and 5 by using the following abbreviations: MA for Mirhassani and Abolghasemi [21], B for Brandão [14], PR for Pisinger and Ropke [45], LGW for Li et al. [46] and FOH for Fleszar et al. [20]. Results from Tables 3–5 indicate that the proposed CW can find high quality solutions within reasonable time, especially for small and medium scale problems. Out of 62 problems, we find the optimal solutions for 60 problems with up to 134 customers. For two problems with 100–134 customers, the percentage deviations between our solutions and the optimal solutions are very low (E-n101-k8 and F-n135-k7). Nevertheless, in those optimal and near optimal solutions, there are four problems (A-n34-k5, P-n50-k7, F-n72-k4, and E-n76-k10) for which our results in Tables 4 and 5 are better than the others. Moreover, our results in Table 3 show that the proposed CW always performed better than CW. These indicate that the proposed CW is effective and efficient in producing high quality solutions for well-known benchmark problems. The important details of our improvement are discussed below.

The average percentage improvements between CW solutions and our solutions for benchmark of data sets A, B, P, E, and F are presented in Table 3. We have found that CW solutions were improved by the average of 15.712%. This finding shows that data set F has the highest average improvement and data set B has the lowest average deviation. The greatest improvements of CW solutions are, respectively, presented in top three instances including A-n39-k6 (26.019%), A-n54-k7 (25.458%), and F-n135-k7 (24.916%). We can conclude that the problems which have the features like clustered customers can be solved by CW better than the problems which have the features like dispersed customers. In addition, the results show that the proposed CW can solve both above-mentioned problems to obtain optimal or near optimal solutions.

The performance of route postimprovement procedure can be described as the reduction of the percentage deviation between CW-2 and CW-3 solutions. The average reduction of the percentage deviation between CW-2 solutions and CW-3 solutions for benchmark of data sets A, B, P, E, and F are 3.652, 3.857, 3.938, 3.699, and 4.806. We have found that CW-3 solutions were reduced by the average of 3.838%. The greatest reductions of CW-3 solutions are, respectively, presented in top three instances including B-n64-k9 (11.769%), P-n50-k7 (10.866%), and A-n45-k6 (10.460%). According to Table 3, some CW-3 solutions can be reduced to obtain the optimal solutions.

Another finding from our work is the infeasible solutions produced by CW in which the number of vehicles used is inadequate. This finding is referred to Vigo [47] that CW does not allow for the control of the number of routes of the final solution. The solution found for a given instance can, in fact, require more than *k* routes to serve all the customers, hence being infeasible.

## 4. Conclusions

In this paper, we have presented a new heuristic approach based on Clarke-Wright algorithm to solve the open vehicle routing problem (OVRP). We have modified the Clarke-Wright algorithm with three procedures composed of Clarke-Wright formula modification, open-route construction, and two-phase selection and have combined them with a route postimprovement procedure in which the neighborhood structures composed of shift, swap, and *λ*-opt move operators are used to improve our best solution. We also have done experiments using six well-known data sets of OVRP (composed of 62 instances) obtained from the literatures. The numerical results show that our approach is competitive and our solutions outperform Clarke and Wright [22] in all directions. Moreover, it also generates the best known solutions in 97% of all instances (60 out of 62).

During the development of our approach, we have mentioned the ideas related to the CW that deserve more attention in further studies. Consequently, it may be interesting to develop a more powerful postimprovement procedure. An additional study is to extend the proposed CW to deal with other variants of the studied problems such as simultaneous pickup and delivery (VRPSPD) or time windows (VRPTW).

## Figures and Tables

**Table 1:** The parameters used in the proposed CW.

Parameter	Values
*The Clarke-Wright formula modification procedure *	
Route shape (*λ*)	0.1–2.0 (increment of 0.1)

*The two-phase selection procedure *	
Number of tournament sizes	3–20 (random number)
Number of global iterations for two-phase selection	5,000

*The route postimprovement procedure *	
Number of consecutive iterations without any improvements in the best found solution	500
Probability to select each move operator	0.125

**Table 2:** The different features of the proposed CW.

Abbreviation	Details
CW-1	Improve CW solution with Clarke-Wright formula modification procedure
CW-2	Improve CW-1 solution with two-phase selection procedure
CW-3	Improve CW-2 solution with route post-improvement procedure

**Table 3:** Computational results between the proposed CW and CW for data sets A, B, P, E, C, and F.

Number	Instance	Solution					Percentage improvement			CPU time (seconds)
		CW	CW-1	*λ*	CW-2	CW-3	CW-1	CW-2	CW-3	
1	A-n32-k5	612.99	535.92	1.7	493.18	487.31	12.571	19.544	20.503	4.819
2	A-n33-k5	496.61	458.52	1.5	425.54	424.54	7.669	14.311	14.512	5.493
3	A-n33-k6	566.16	546.18	1.7	464.10	462.43	3.528	18.026	18.321	5.289
4	A-n34-k5	*547.27 *	*520.09 *	1.1	519.51	508.17	4.966	5.071	7.143	5.675
5	A-n36-k5	689.48	571.70	1.8	535.23	519.46	17.083	22.373	24.660	6.134
6	A-n37-k5	579.74	541.64	1.9	511.12	486.24	6.572	11.836	16.128	6.689
7	A-n37-k6	*712.86 *	*612.76 *	1.9	616.26	581.07	14.041	13.551	18.487	6.825
8	A-n38-k5	*552.83 *	*520.78 *	1.6	531.49	498.00	5.796	3.859	9.918	7.092
9	A-n39-k5	704.55	*590.71 *	1.2	554.80	549.68	16.158	21.254	21.980	7.568
10	A-n39-k6	720.55	558.86	1.9	547.57	533.07	22.439	24.007	26.019	7.494
11	A-n44-k6	*757.90 *	*724.33 *	1.1	641.77	617.39	4.430	15.323	18.540	10.323
12	A-n45-k6	*734.97 *	*643.56 *	2.0	716.52	648.67	12.437	2.511	11.742	10.414
13	A-n45-k7	829.44	759.40	1.6	699.86	685.16	8.444	15.622	17.395	10.754
14	A-n46-k7	738.00	645.24	1.1	593.57	583.54	12.569	19.570	20.930	11.259
15	A-n48-k7	789.39	756.58	1.1	726.54	669.83	4.156	7.962	15.146	12.534
16	A-n53-k7	*816.38 *	*702.85 *	1.5	665.39	655.18	13.907	18.495	19.746	15.624
17	A-n54-k7	951.51	*808.42 *	1.5	723.60	709.27	15.038	23.952	25.458	16.224
18	A-n55-k9	*802.06 *	*736.91 *	1.9	696.52	669.06	8.123	13.158	16.582	17.324
19	A-n62-k8	*979.51 *	851.15	1.3	815.21	783.18	13.105	16.774	20.044	22.959
20	A-n65-k9	*844.35 *	*800.41 *	1.2	783.42	728.59	5.205	7.217	13.710	24.524
21	A-n69-k9	*942.87 *	*798.64 *	2.0	773.17	757.76	15.296	17.998	19.632	28.600

	Average percentage improvement of data set A						10.644	14.877	17.933	11.601

1	B-n31-k5	383.68	367.00	0.9	364.80	362.73	4.347	4.923	5.463	4.365
2	B-n34-k5	*541.34 *	506.26	0.9	459.59	458.76	6.480	15.103	15.254	5.556
3	B-n35-k5	599.16	595.15	1.4	567.34	557.33	0.669	5.311	6.982	5.981
4	B-n38-k6	500.64	*483.14 *	1.4	450.72	445.63	3.495	9.972	10.989	7.109
5	B-n39-k5	*382.11 *	*354.03 *	1.4	334.70	322.54	7.350	12.409	15.590	7.602
6	B-n41-k6	*539.15 *	*507.25 *	1.8	493.34	483.07	5.917	8.496	10.402	8.549
7	B-n43-k6	483.21	*481.57 *	1.6	432.30	428.17	0.340	10.536	11.391	9.422
8	B-n44-k7	575.52	560.54	1.9	512.64	501.31	2.603	10.927	12.895	10.244
9	B-n45-k5	601.71	*512.73 *	1.9	509.56	488.07	14.788	15.315	18.887	10.357
10	B-n45-k6	*459.90 *	*430.83 *	1.1	431.54	403.81	6.323	6.168	12.197	10.573
11	B-n50-k7	537.23	*491.93 *	1.9	446.07	437.15	8.433	16.969	18.629	13.265
12	B-n51-k7	*703.81 *	*683.31 *	2.0	656.01	625.14	2.913	6.792	11.178	13.509
13	B-n52-k7	482.90	465.17	1.8	450.07	441.19	3.672	6.798	8.637	14.586
14	B-n56-k7	497.15	*474.13 *	1.5	463.06	420.48	4.629	6.856	15.420	17.409
15	B-n63-k10	950.48	950.48	1.0	857.90	837.07	0.000	9.740	11.931	23.724
16	B-n64-k9	*572.41 *	*541.70 *	1.2	581.72	520.47	5.364	−1.627	9.074	23.764
17	B-n68-k9	830.48	*777.68 *	1.1	758.69	701.72	6.357	8.644	15.504	27.874

	Average percentage improvement of data set B						4.922	9.020	12.378	12.582

1	P-n16-k8	235.89	235.89	0.3	235.06	235.06	0.000	0.352	0.352	1.173
2	P-n19-k2	*198.25 *	*172.93 *	1.9	168.57	168.57	12.771	14.972	14.972	1.565
3	P-n20-k2	210.01	*184.50 *	1.9	170.28	170.28	12.147	18.918	18.918	1.712
4	P-n21-k2	209.92	180.27	1.8	168.15	163.88	14.124	19.897	21.933	1.729
5	P-n22-k2	206.00	183.58	1.8	171.46	167.19	10.883	16.765	18.840	1.888
6	P-n22-k8	*370.26 *	*345.53 *	1.6	352.14	345.87	6.679	4.892	6.588	2.330
7	P-n23-k8	*309.74 *	*307.28 *	1.7	304.83	302.87	0.794	1.586	2.219	2.608
8	P-n40-k5	420.65	395.73	1.5	370.64	349.55	5.924	11.889	16.902	7.749
9	P-n45-k5	*459.29 *	442.23	1.5	396.64	391.81	3.714	13.641	14.692	10.159
10	P-n50-k7	468.42	447.69	1.5	440.56	397.38	4.426	5.948	15.167	13.237
11	P-n55-k7	513.45	464.90	1.4	452.69	411.58	9.455	11.833	19.840	16.627
12	P-n55-k8	505.72	476.43	1.8	442.21	412.55	5.793	12.558	18.423	16.349
13	P-n55-k10	555.25	*502.56 *	1.6	488.65	444.31	9.490	11.995	19.981	17.387
14	P-n60-k10	*584.40 *	*539.43 *	1.8	503.38	482.09	7.694	13.864	17.507	20.499
15	P-n65-k10	*649.31 *	592.35	1.6	531.57	522.50	8.772	18.133	19.529	29.626

	Average percentage improvement of data set P						7.511	11.816	15.058	9.642

1	E-n22-k4	286.91	260.61	1.3	252.61	252.61	9.169	11.955	11.955	3.753
2	E-n23-k3	497.18	456.86	1.2	444.29	442.98	8.111	10.638	10.901	4.156
3	E-n33-k4	633.04	576.29	1.4	518.04	511.26	8.965	18.165	19.236	12.223
4	E-n51-k5	*493.02 *	*477.78 *	2.0	452.67	416.06	3.091	8.185	15.610	36.154
5	E-n76-k10	*697.03 *	*641.48 *	1.9	587.35	567.14	7.971	15.736	18.635	68.917
6	E-n101-k8	807.33	724.48	2.0	694.88	642.36	10.262	13.929	20.433	82.387

	Average percentage improvement of data set E						7.928	13.101	16.128	34.598

1	F-n45-k4	*615.12 *	535.85	2.0	478.40	463.90	12.886	22.227	24.584	10.365
2	F-n72-k4	*208.29 *	*191.18 *	2.0	187.67	177.00	8.212	9.899	15.021	42.554
3	F-n135-k7	*1033.24 *	*856.33 *	1.9	816.30	775.80	17.122	20.996	24.916	112.258

	Average percentage improvement of data set F						12.740	17.707	21.507	55.059

Italic number indicates the infeasible solution (the number of vehicles used is inadequate).

**Table 4:** Computational results between the proposed CW and MA for data sets A, P, and F.

Number	Instance	Solution			Percentage deviation	
		Best Known	MA	Our CW	MA	Our CW
1	A-n32-k5	487.31	487.31	487.31	0.000	0.000
2	A-n33-k5	424.54	424.54	424.54	0.000	0.000
3	A-n33-k6	462.43	462.43	462.43	0.000	0.000
4	A-n34-k5	508.17	508.52	508.17	0.068	0.000
5	A-n36-k5	519.46	519.46	519.46	0.000	0.000
6	A-n37-k5	486.24	486.24	486.24	0.000	0.000
7	P-n19-k2	168.57	168.57	168.57	0.000	0.000
8	P-n20-k2	170.28	170.28	170.28	0.000	0.000
9	P-n21-k2	163.88	163.88	163.88	0.000	0.000
10	P-n22-k2	167.19	167.19	167.19	0.000	0.000
11	P-n40-k5	349.55	349.55	349.55	0.000	0.000
12	P-n45-k5	391.81	391.81	391.81	0.000	0.000
13	P-n50-k7	397.38	407.73	397.38	2.605	0.000
14	F-n45-k4	463.90	463.90	463.90	0.000	0.000
15	F-n72-k4	177.00	177.45	177.00	0.257	0.000

	Average percentage deviation of data sets A, P, and F				0.195	0.000

**Table 5:** Computational results between the proposed CW and the other algorithms for data sets E and F.

Number	Instance	Solution					
		Best Known	B	PR	LGW	FOH	Our CW
1	E-n51-k5	**416.06**	**416.1**	**416.06**	**416.06**	**416.06**	**416.06**
2	E-n76-k10	**567.14**	574.5	**567.14**	**567.14**	**567.14**	**567.14**
3	E-n101-k8	**639.74**	641.6	641.76	**639.74**	**639.74**	642.36
4	F-n72-k4	**177.00**	177.4	**177.00**	**177.00**	178.09	**177.00**
5	F-n135-k7	**769.66**	781.2	770.17	**769.66**	**769.66**	775.80

Number	Instance		CPU time (seconds)				
			B	PR	LGW	FOH	Our CW

1	E-n51-k5		88.8	230	6.2	17.6	36.154
2	E-n76-k10		167.5	530	31.3	29.0	68.917
3	E-n101-k8		325.3	1280	39.5	239.6	82.387
4	F-n72-k4		398.1	1040	19.5	140.2	42.554
5	F-n135-k7		1000.2	3590	158.2	1237.5	112.258

Bold number indicates the best known solution was obtained.
